# Setting incentives right with long-term risk adjustment

**DOI:** 10.1007/s10198-024-01751-6

**Published:** 2024-12-20

**Authors:** Simon Reif, Sabrina Schubert, Achim Wambach

**Affiliations:** 1 ZEW – Centre for European Economic Research, L7 1, 68161 Mannheim, Germany; 2 FAU Erlangen-Nürnberg, Nürnberg, Germany; 3 University of Mannheim, Mannheim, Germany

*Current risk adjustment schemes in social health insurance provide little incentives for health insurers to engage in investments in enrollee health and efficiency enhancing innovations. We propose a long-term risk adjustment scheme*,* which still prevents risk selection*,* but makes it financially attractive for insurers to care about long-term health of their enrollees and increases efficiency.*

## The role of risk adjustment in social health insurance

A key challenge for health policy is to offer affordable health insurance premiums to a population characterized by large heterogeneities in risks and costs. Many health systems have decided not to set premiums based on the individual enrollee risk, but instead aim for premium equalization across the population. This equalization creates incentives to select low-risk (and therefore low cost) individuals. To deter insurances from discriminating against individuals with high expected healthcare costs, many social health insurance systems (e.g. Belgium, Germany, Switzerland, the Netherlands or the US Medicare and Medicaid) have introduced morbidity-based risk adjustment schemes. Under these schemes, insurances receive the expected healthcare costs for each insured individual.

In the ideal case, the risk adjustment scheme makes every enrollee equally attractive to the insurance, allowing them to focus on cost management and service quality instead of selecting good risks. Most risk adjustment schemes have started with baseline setups where only a small set of variables such as region, gender and age are used as risk adjusters [8]. Today, most schemes are more elaborated and in particular also take morbidity data into account. These advances have increased the accuracy of determining expected health costs. The focus of many academic contributions in the area of risk adjustment has been the technical design to optimize the accuracy of risk adjusted payments using classical statistics [5, 7, 10, 13], and more recently machine learning [3, 4, 9, 14].

## Incentives for investing in enrollee health

Risk adjustment, which reduces incentives for the insurance to discriminate against high-cost individuals, mimics the premiums in a competitive insurance market: The overall payments received by insurances, i.e. the expected costs of the insured, coincide with the fair premium for one-year insurance contracts. However, Eggleston et al. [1] show in a theoretical model that risk adjustment reduces investment in preventive activities. The intuition behind the model is that when insurances receive expected healthcare costs estimated from morbidity data, they lack incentives to mitigate morbidity related cost increases through (costly) prevention.

The general pattern that insurances invest in the health of their enrollees only when it pays off financially can be observed in the private employer-sponsored US health insurance market without risk adjustment. An empirical example highlighting the negative effect of risk adjustment on prevention comes from a reform of the Medicare Advantage scheme in the US. Lissenden & Balkrishnan [6] show that when diagnoses (including those preventable by vaccinations) are added to risk adjustment, vaccination rates go down. Weinhold et al. [15] show that in the German social health insurance market, expenditures on prevention result in a net loss for insurances. When considering the change in risk adjustment payment for the then healthier individual in this setting, the costs of investing in successful prevention outweigh the savings. In their analysis of US health insurances, Fang & Gavazza [2] do however show that healthcare investment is higher when the expected tenure of the employee (at the firm and therefore also at the health insurance) is longer. This indicates that – given the right long-term incentives – insurances are willing to invest in long-term health.

While empirical evidence on negative incentives for health insurances focuses on preventive activities, the general pattern also applies to other settings. Decisions regarding investment in enrollee health also matter when curative therapies (e.g. Directly Acting Antivirals for Hepatitis C or CFTR modulators for Cystic Fibrosis) are more favorable in the long run than maintenance therapies. Other examples concern the investment in costly infrastructure (e.g. patient monitoring or rehabilitation facilities) which increase immediate costs and will only yield benefits by reduced expected costs in the future.

## A simple example of incentives in risk adjustment schemes

As explained above, the financial stream to the health insurance following risk adjustment schemes follows the same principles as a one-year contract in a competitive insurance market: Each insurance receives the expected expenditures of their enrollees for the following year. These annual payments facilitate efficient resource management for the upcoming year. However, annual payments from the risk adjustment scheme imply that insurances have a financial incentive to invest in enrollee health only if the investment pays off within a year.

**Fig. 1:**
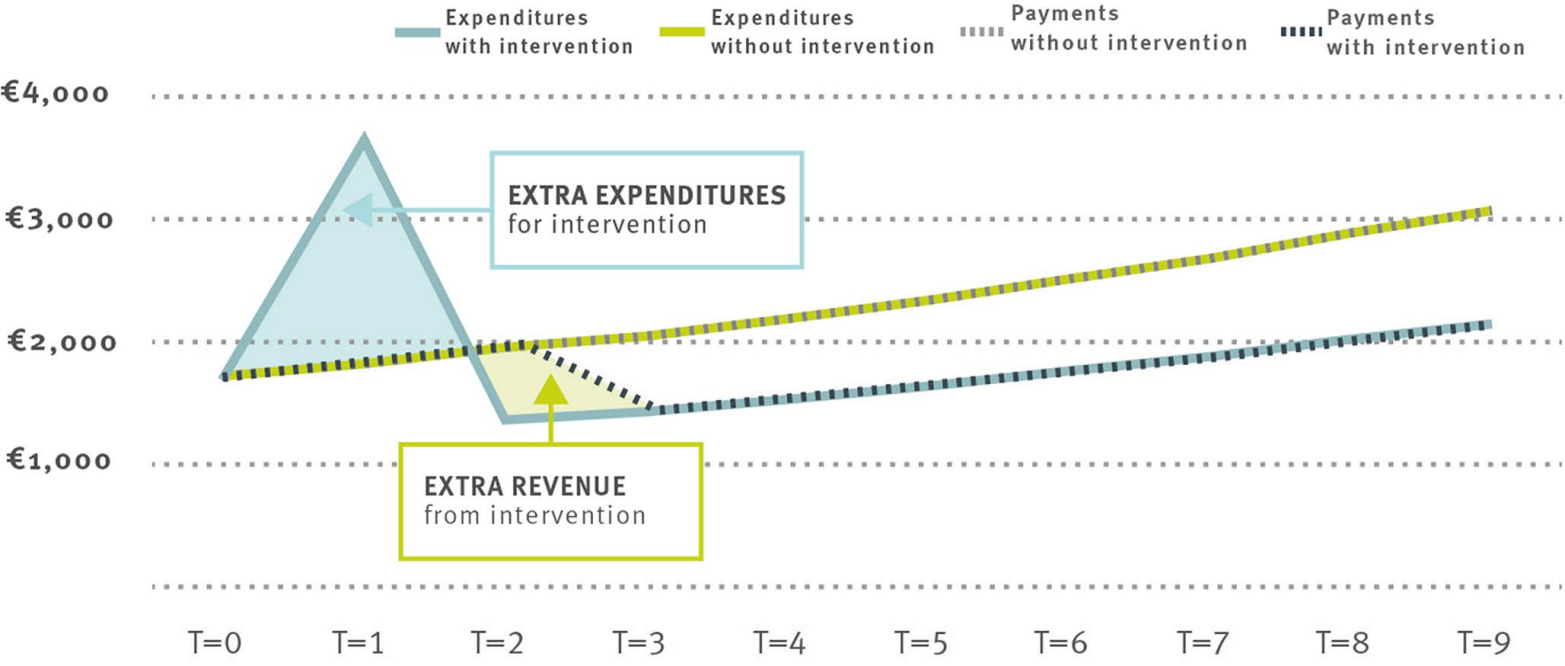
Illustration of one-year risk adjustment scheme

Figure 1 illustrates these incentives. The calculations are based on the average expenditures in the German social health insurance system from the year 2021. We assume that the allocation and the expected expenditures for a 40-year-old man are 1,720 euros in the first year. The allocation in the following years increases due to two factors: First, an assumed health cost inflation rate of 3% per year (overall inflation is ignored to make payments comparable across time). Second, the expected costs in the second year are those of a 41-year-old, in the third year those of a 42-year-old, etc. The green line depicts this cost progression, along with the corresponding annual payments from the risk adjustment scheme to the insurance. The blue line represents a potential expenditure curve when the insurance finances an intervention in T = 1 that costs twice the expected expenditures in the intervention year compared to normal treatment, but saves 30% of the originally expected costs in the long run. Note, that these cost savings will be reflected in the annual payment to the insurance, shown by the dotted black line: The intervention improved the health of enrollee and the risk adjustment scheme reduces the payments to the insurance. Without the intervention, the insurance receives total payments of 23,211 euros over ten years and incurs expenditures of the same amount. If the intervention is financed, total costs over a ten-year horizon are lower at 19,132 euros (due to reduced expenditures from year 2 onwards), but the total payments from the scheme are only 17,896 euros (since the expected costs and therefore payments to the insurance are reduced from year 3 onwards). Therefore, the insurance has no incentive to implement measures that only yield benefits in subsequent years.

## Proposed measures to incentivize prevention under risk adjustment

The lack of incentives for prevention has initiated a debate on possible health system reforms. Currently, there are three approaches to modify the incentive structure for investing in enrollee health in health insurance systems. First, one option is reverting to simpler risk adjustment (partly) ignoring diagnoses: While this would increase the incentive to invest in prevention, it can also lead to stronger incentives for risk selection based on diagnoses. Second, policymakers could mandate insurances to invest directly in prevention. This approach is for example applied in the German social health insurance. While such mandates increase preventive activities, they are unlikely to result in optimal prevention as there is still no incentive to provide the most effective interventions. Additionally, this strategy does not consider further investments in enrollee health, as discussed above. A third way proposed by Eggleston et al. [1], is to apply pay-for-performance for preventive activities. The idea is to financially reward insurances when the health state of their enrollees developed better than predicted, setting a direct incentive to invest in their health. While such a scheme would address the incentives for prevention, operationalizing it is challenging due to the possibility of enrollees having very specific combinations of diagnoses that interact in their effect on health status.

## New proposal: Setting long-term incentives in risk adjustment

**Fig. 2:**
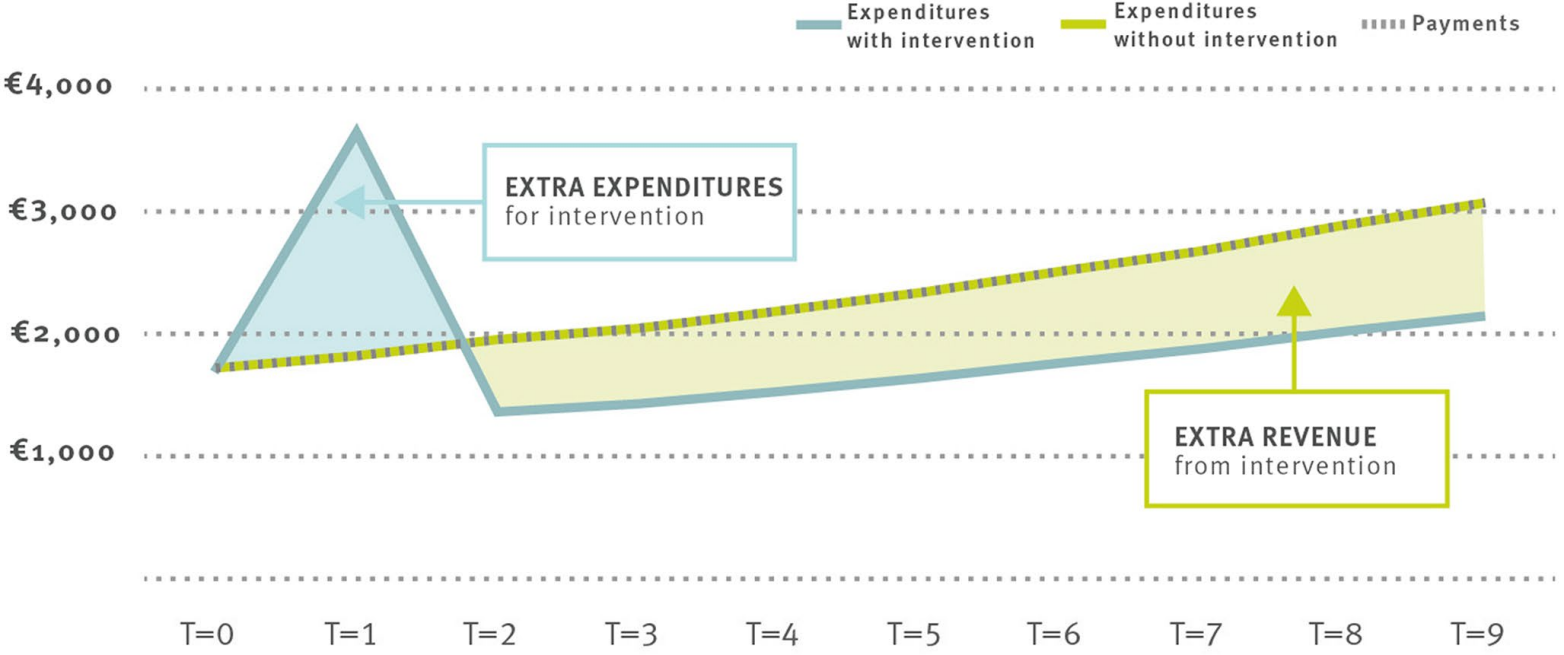
Illustration of ten-year risk adjustment scheme

We propose a novel approach to reward insurances for successful investment in enrollee health. Building on the idea from Eggleston et al. [1], we propose to break the nexus between health improvements and lower allocations. We suggest that longer time periods in the risk adjustment distribution mechanism make investments in prevention and innovation attractive for health insurances. The long-term nature can be achieved by changing the time frame of the payment calculations. Instead of annual calculations, the expected expenditures for the enrollees should be computed for the following ten years based on current enrollee characteristics. Each year, the insurance would then receive the initially calculated annual amount for the enrollee for the respective year. From an insurance standpoint, this corresponds to a shift from a one-year-contract towards a ten-year contract. Through this longer-term calculation of payments, health insurances can benefit from cost savings resulting from improved treatment. A healthier enrollee incurs lower expenditures in the following years, while the payments to the insurance remain unchanged – thereby, financially benefiting the insurance. On the other side, a sicker enrollee causes higher expenses than the payments from the scheme, causing a financial loss. The insurance is therefore incentivized to invest in the long-term health of its enrollees. At the same time, the ten-year calculation of allocations ensures that, on average, an insurance does not incur losses. The allocations exactly correspond to the “fair premium” that would be demanded in a competitive market with ten-year contracts. The mechanism of such as scheme is shown in Fig. 2.

In contrast to Fig. 1, payments from the risk adjustment schemes are not adjusted after a successful intervention. Therefore, the insurance now has total expenditures of 19,132 euros over the ten-year period but receives a total a surplus of 4,080 euros since the cost savings remain with the insurance. This surplus comes with two positive effects. Firstly, the surplus can be used to implement care innovations that further reduce future health expenditures. Secondly, the allocations, based on average costs for all insurances, decrease – thereby reducing the overall cost of the healthcare system.

## Practical considerations for a long-term risk adjustment scheme

(i) Switching insurance One big advantage of the current risk-based adjustment scheme is that it allows enrollees to switch insurance on an annual basis. This advantage remains with the long-term risk adjustment scheme, if switches happen according to the following rules. Suppose an enrollee decides to switch from insurance A to insurance B after three years, so her initial risk assessment at insurance A was made three years ago. Upon becoming a policyholder of insurance B, she will receive a new ten-year assessment forming the basis for the payments to insurance B. If the expected costs have increased in this new assessment, the departing insurance A must compensate the difference to the risk adjustment scheme for the years the enrollee would have otherwise remained with it, in the example for seven years. If the expected costs have meanwhile fallen, insurance A receives the difference for these seven years from the scheme. Thus, the switch of an enrollee to another insurance is in expectation financially neutral for both the departing insurance A and the receiving insurance B.

(ii) High-cost patients The reassessment of risk for individuals switching between insurances also alleviates the threat of discrimination against individuals with adverse health events in the first years of the ten-year period. Although such an individual is then loss-making for the insurance, the insurance must decide between covering the costs for treatment or compensate the loss after reassessment when the individual switches insurance. Conceptually, such a discrimination risk can be further reduced with dedicated mechanisms for extreme-cost patients such as risk sharing [12] or high-risk pooling [11].

(iii) End of life The proposed mechanism relies on the assumption that enrollees with better health are less costly, as investments in health lead to lower expenditures in the future. However, this assumption may not always hold true. Particularly for end-of-live spending, health benefits and financial benefits might diverge. To avoid problematic incentives (beyond the classic risk adjustment scheme), the calculation period of the proposed risk adjustment scheme could be reduced from ten to five (one) years for individuals older than 70 (80).

(iv) Challenge of long-term cost prediction Much of the recent technical literature on risk adjustment focuses on methods to improve the predictions of the risk adjustment models. While precisely predicting health care costs is challenging for the currently common one-year schemes, extending the prediction period will cp. worsen the fit. However, as long as the prediction error is not correlated with information observable to the insurance, a ten-year prediction period does not increase selection incentives. Better data and more advanced statistical models can also alleviate this concern in the future. The threads from prediction error can be further reduced by separate financing mechanisms for extreme-cost patients and the adjustment of the calculation period to fewer years for older individuals which have a higher variance in costs.

## Conclusion

While morbidity-based risk adjustment strongly decreases the incentive for insurance to discriminate against enrollees with high expected costs, the current one-year timeframe for payment calculation and allocation binds insurances to short-term decision-making regarding enrollee health investments. Consequently, insurances have weak incentives to invest in prevention and in innovative technologies, especially costly interventions that only financially pay off in later years. We propose extending the risk adjustment allocation period to span over ten years, enabling insurances to benefit from investment in enrollee health. In particular in increasingly common managed care plans, insurances can better use long-term investments to benefit their finances, enrollee health and ultimately increase the financial sustainability of healthcare systems.
